# Factors Associated with Acute Kidney Injury among Children with Severe Malaria at Kiryandongo General Hospital, Uganda

**DOI:** 10.1155/2023/2139016

**Published:** 2023-07-08

**Authors:** Lokengama Kwambele, Grace Ndeezi, Yamile Arias Ortiz, Sabinah Twesigemuka, Martin Nduwimana, Walufu Ivan Egesa, Patrick Kumbowi Kumbakulu, Yves Tibamwenda Bafwa

**Affiliations:** ^1^Department of Paediatrics and Child Health, Faculty of Clinical Medicine and Dentistry, Kampala International University, Uganda; ^2^Department of Paediatrics and Child Health, Faculty of Clinical Medicine and Dentistry, Makerere University, Uganda; ^3^Nile International Hospital, Jinja, Uganda; ^4^Department of Internal Medicine, Faculty of Medicine, University of Bunia, Democratic Republic of the Congo

## Abstract

**Background:**

Malaria remains one of the leading health problems of the developing world, and acute kidney injury (AKI) is a well-recognized complication of severe malaria in adults; but the clinical importance of AKI in paediatric severe malaria is not well documented. Knowledge of the prevalence and factors associated with AKI among children with severe malaria is among the key strategies, which can help to reduce the burden of AKI among this vulnerable group. *Methodology*. A hospital-based prospective cross-sectional descriptive and analytic study of children with severe malaria was carried out at Kiryandongo General Hospital. The study involved 350 children with severe malaria attending the study site from August to October 2021. Questionnaires were administered to caretakers to obtain sociodemographic characteristics. Medical data were obtained through physical examination followed by laboratory tests. Blood samples were tested for creatinine and blood smear for malaria. Data were analyzed using binary logistic regression (bivariate and multivariate) to assess for the factors associated with AKI. A *p* value < 0.05 was considered statistically significant.

**Results:**

The mean age of children with severe malaria was 7.0 ± 3.8 years, and 54.3% of them were male. Of the 350 children enrolled, 167 had AKI, giving an overall AKI prevalence of 47.7% (95% CI: 42.5-53.0). The factors that were significantly associated with AKI among children with severe malaria included caretaker with no formal education (aOR = 21.0, 95% CI: 1.68–261.18, *p* = 0.018), caretaker with primary education level (aOR = 4.5, 95% CI: 1.41–14.12, *p* = 0.011), age of child < 5 years (aOR = 1.8, 95% CI: 1.07–2.88, *p* = 0.025), history of receiving NSAIDs (aOR = 5.6, 95% CI: 2.34–13.22, *p* < 0.001), moderate anemia (aOR = 3.1, 95% CI: 1.39–6.94, *p* = 0.006), and severe anemia (aOR = 3.8, 95% CI: 1.66–8.55, *p* = 0.002).

**Conclusion:**

The prevalence of AKI was high among children with severe malaria in Kiryandongo General Hospital. Acute kidney injury among children with severe malaria was associated with low level of education of caretakers, age of children less than 5 years, history of receiving NSAIDs, and anemia. The management of severe malaria should include screening for AKI especially in children under five years of age, anemic, and those who have received NSAIDs.

## 1. Introduction

Acute kidney injury has become increasingly prevalent in both developed and developing countries and is associated with severe mortality and morbidity especially in children. Acute kidney injury (AKI) in developed countries occurs predominantly in urban area (intensive care unit), and it is associated with multiorgan failure and sepsis with high mortality in older population, while AKI in rural region commonly develops in response to a single disease like malaria and in younger healthy children [1]. In Uganda, malaria is still a major public health problem associated with low socioeconomic development and poverty and the most reported disease at both public and private health facilities [2]. Children, especially those under the age of 5 years, are among the most prone to malaria infection, as they have not yet developed any immunity to the disease.

In tropical regions, malaria was the first parasitic infection to be clearly associated with glomerular diseases [3]. Several mechanisms explain the pathophysiological mechanism underlying AKI in severe malaria. Severe malaria can cause disease in glomeruli, tubules, and in the interstitial region. Kidney disease in malaria is primarily due to erythrocyte abnormalities. Parasitized red cells tend to adhere to healthy erythrocytes, blood platelets, and capillary endothelium, leading to formation of rosettes and clumps, which impair microcirculation [4], and these events are probable contributing factors for kidney injury, in association with hemodynamic instability, including hypovolemia and shock. Endothelial activation leads to the release of several cytokines, including thromboxane, catecholamines, endothelin, and other inflammatory mediators that are also implicated in the pathogenesis of malaria-associated kidney injury [5].

The prevalence and clinical importance of AKI among children with severe malaria (SM) are not well documented in some high malaria endemic areas. There is evidence that even small changes in kidney function are associated with increased morbidity, mortality, and the risk of developing chronic kidney disease (CKD) in children with severe malaria [6]. Previous studies conducted in Uganda have reported a high prevalence of AKI among children with severe malaria ranging from 35.1% to 45.5% [6, 7].

Kiryandongo is among districts with a high prevalence of malaria in Uganda with 214/1000 confirmed malaria cases despite the effort of the government and the sensitization from the local authority on the use of mosquito nets [8]. According to health statistics of Kiryandongo District Local Government (KDLG) in 2017, malaria was the leading cause of morbidity. Kiryandongo has a hot climate which is favorable for the life cycle of plasmodium, and it has the biggest refugees' camp of the country with a low socioeconomic status. There is no data related to the prevalence of AKI, and factors associated with AKI in this geographic area are unknown. Hence, this study seeks to bridge that gap by determining the prevalence and factors associated with AKI among children with severe malaria in Kiryandongo General Hospital.

## 2. Methods

### 2.1. Study Design

This was a prospective hospital-based cross-sectional descriptive and analytical study to determine the prevalence and factors associated with AKI among children with severe malaria in Kiryandongo General Hospital.

### 2.2. Study Site

The study was conducted at Kiryandongo General Hospital which is located in Kikube Parish, Kiryandongo subcounty, Kibanda County, in Kiryandongo District, about 50 kilometers (31 miles), northeast of Masindi General Hospital. This is approximately 211 kilometers (131 miles) north of the Mulago National Referral Hospital. Kiryandongo General Hospital is a 109-bed government-owned hospital. It serves Kiryandongo District and parts of the districts of Masindi, Nakasongola, Oyam, Apac, Amuru, and Nwoya. The paediatric department has 50 beds. The hospital has a laboratory which can make the diagnosis of acute kidney injury and other chemistry investigations.

### 2.3. Study Population

The study population included all children from 2 months to 12 years admitted with severe malaria at Kiryandongo General Hospital both in paediatric ward and emergency department. This study included all children aged 2 months to 12 years and admitted with severe malaria after obtaining informed consent from caretaker and assent from children above 8 years. Children with malnutrition, preexisting kidney diseases, and conditions that cause chronic kidney injury such as hypertension, sickle cell disease, and chronic glomerulonephritis were excluded. The sample size was 350 and was calculated using the Kish Leslie formula [9] based on a previous prevalence of AKI of 35.1% reported by Conroy et al. in Uganda [6]. Participants were consecutively enrolled until the target number was attained. Study participants were selected as they come to the outpatient and emergency departments. Every patient meeting the selection criteria was eligible to participate.

### 2.4. Data Collection

A structured questionnaire was administered by the investigator using English or Runyoro according to the preference of the participant. It was a face-to-face interview which allowed clarification. The questions were open and close ended. Variables included sociodemographic, medical, and laboratory factors. Sociodemographic factors included caretakers' sociodemographic factors (age and level of education) and child's sociodemographic factors (age and sex). Medical factors included impaired consciousness or unarousable coma, generalized body weakness, prostration or lethargy (child is unable to feed, to walk, or to sit up without assistance), multiple convulsions (more than 2 episodes in 24 h), respiratory distress (acidotic breathing), clinical jaundice plus evidence of other vital organ dysfunction, dehydration, and previous medication. Dehydration was classified as no dehydration, some dehydration, and severe dehydration. The laboratory factors included hypoglycemia (blood glucose < 2.2 mmol/L or <40 mg/dL). Anemia was defined as a Hb < 11 g/dL and was further classified as mild anemia (11 g/dL), moderate anemia (Hb < 8 g/dL), and severe anemia (Hb < 5 g/dL) [10]. Hyperparasitemia was defined by presence of malaria parasites 1-10/single high-power field (3+), or >10/high-power field (4+). Acute kidney injury was defined by a serum creatinine > 265 micromol/L. Acute kidney injury was further classified in 3 grades: grade 1 was defined as 1.5-1.9 times increased in SCr from the baseline OR ≥ 0.3 mg/dL increase; grade 2 referred to an increased by 2.0-2.9 times baseline; grade 3 was defined by a 3.0 times increase from the baseline, OR SCr ≥ 4.0 mg/dL, and OR initiation of renal replacement therapy, OR eGFR < 35 mL/min per 1.73 m^2^. The physical examination was conducted to assess the presence of the following signs: alteration of the level of consciousness, jaundice, pallor, degree of dehydration, cyanosis, temperature and respiratory rate, oxygen saturation, and organomegaly, to mention but a few. Full blood count was analyzed using Sysmex Automated Hematology Analyser at Kiryandongo GH. Blood glucose was measured using FreeStyle Optium Glucometer by the principal investigator at admission. Thick blood smears for malaria parasites were done using the Field stains A and B and examined by a laboratory technician using the microscope in the main hospital laboratory. Serum creatinine was done for the diagnosis of acute kidney injury. Blood sample was drawn during insertion of intravenous cannula for measurement of full blood count, blood glucose, and blood creatinine. Prior to drawing of blood, the area was swabbed with cotton dipped in ethyl alcohol 70% to prevent contamination. Five milliliters of blood was drawn for determination of full blood count, blood glucose, and serum creatinine on admission. The red vacutainer was used to collect 3 mL of blood for serum creatinine test, and 2 mL of blood was collected in the purple vacutainer for CBC and BS test. These laboratory investigations were done on admission in the private laboratory of Kiryandongo Referral Hospital. Severe malaria was confirming by thick field smear of 1-10 parasites per thick field (+3) or of 10 or more parasites per thick field (+4).

Malaria was defined by the presence of malaria parasites on blood smear. Severe malaria was defined as malaria plus any of the following complications: impaired consciousness (defined as prostration (generalized weakness so that the patient is unable to walk or sit up without assistance) or coma (an unrousable state with a corresponding Blantyre Coma Scale (BCS) ≤ 2) where no other cause other than malaria could be identified), multiple convulsions, acidosis, hypoglycemia, severe anemia, oliguria, jaundice, pulmonary edema, spontaneous bleeding, hemoglobinuria, shock, and hyperparasitemia [10].

### 2.5. Data Analysis

All statistical analyses were performed with STATA version 14.2. Continuous data are presented as the mean ± standard deviation (SD), and categorical variables are summarized as frequency and percentage. The proportion of AKI among children with severe malaria was summarized as frequencies and percentage. The outcome of children with AKI was determined as a proportion of mortality/discharge among all children with AKI. Factors associated with AKI among children with severe malaria were determined using logistic regression. In bivariate analysis, based on both chi-square test and logistic regression, repeated analysis comparing each independent variable with AKI was done. Crude odd ratio (cOR) with their corresponding 95% CI and *p* values was reported. A variable was considered significant if it has a *p* value < 0.05. All factors with a *p* value < 0.05 or with a borderline *p* value < 0.2 and those which were biologically plausible with AKI among children with severe malaria were considered in the multivariate analysis which was performed to control confounding. The factors in the final multivariate model were reported together with their adjusted odd ratios and 95% CI with their respective *p* values. A variable was considered significant in this analysis if it had a *p* value < 0.05.

## 3. Results

During the study period, 797 children were seen in KGH. Among 469 who had a positive blood smear for malaria, 358 had severe malaria. Eight children were excluded, and the rest were consecutively enrolled. Among the excluded, four refused to consent, three died before full assessment, and one was discharged against medical advice. Among the 350 children with severe malaria, 167 (47.7%) had AKI.

### 3.1. Baseline Characteristics of Study Respondents

The enrolled children had a mean age of 7.0 years (SD: 3.8), with majority aged between 5 and 12 years (68.9%). There were slightly more males (54.3%) than females. The mean age of their caretaker was 28.8 years (SD: 7.4) with majority at least 20 years (84.3%) and with primary education (92.3%). The children had a mean duration of illness of 3.1 ± 0.93 days before admission with majority reporting to hospital after at least 3 days of illness (82.0%), with hyperparasitemia (76.3%) and with about half having moderate to severe anemia (51.7%) (Table 1).

#### 3.1.1. Main Symptoms of Children with Severe Malaria at Admission

**Figure 1:**
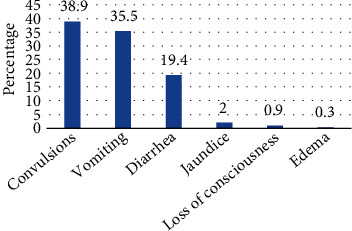
Main symptoms.

The commonest symptoms of children with severe malaria were convulsions (38.9%), vomiting (35.5%), and diarrhea (19.4%) (Figure 1).

### 3.2. Prevalence of Acute Kidney Injury among Children with Severe Malaria

**Figure 2:**
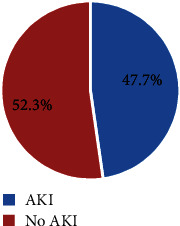
Prevalence of acute kidney injury among children with severe malaria at KGH.

Of the 350 children enrolled in the study with severe malaria, 167 had AKI, giving an overall AKI prevalence of 47.7% (95% CI: 42.5-53.0). Of these, 99 (59.3%), 57 (34.1%), and 11 (6.6%) had grade 1, 2, and 3 AKI, respectively (Figure 2).

### 3.3. Factors Associated with Acute Kidney Injury among Children with Severe Malaria

In multivariate analysis, the factors that were independently associated with AKI among children with severe malaria were caretaker with no or primary education, age of child < 5 years, history of receiving NSAIDs, and having severe anemia. The odds of AKI were 21 and 4.5 times higher among children whose caretakers had no formal level of education or primary level of education, respectively, as compared to those with secondary education. The odds of AKI were 1.8 times higher among children aged less than 5 years as compared to those aged 5 years and above (OR = 1.8, 95% CI: 1.07-2.88, *p* = 0.025). The odds of AKI were 5.6 times higher among children with history of receiving NSAIDS as compared to those with negative history of NSAIDS. The odds of AKI were 3.1 and 3.8 times higher among children with moderate and severe anemia as compared to those with normal hemoglobin (Table 2).

## 4. Discussion

This was a hospital-based cross-sectional descriptive and analytical study to determine the prevalence and factors associated with acute kidney injury among children with severe malaria at Kiryandongo General Hospital, Uganda.

### 4.1. Prevalence of AKI among Children with Severe Malaria at Kiryandongo General Hospital

The prevalence of AKI among children with severe malaria in this study was 47.7% (Figure 2). The prevalence of AKI was higher compared to the findings of most previous studies. In Uganda, two studies have been conducted in Jinja and Mulago by Conroy et al. [6, 7] reporting an overall prevalence of AKI among children with severe malaria of 45.5% and 35.1%, respectively. Similarly, a cross-sectional study conducted in Nigeria by Kunuanunua et al. [11] reported a prevalence of 38%. Through a follow-up study conducted in Democratic Republic of Congo, Marzuillo et al. reported a prevalence of 23.6% [12]. The discrepancy could be related to the fact that Kiryandongo District is one of the high transmission districts with high prevalence of malaria as compared to the low transmission district such as Kampala and Jinja. Kiryandongo has a hot climate which is favorable for the life cycle of the plasmodium, and it has the biggest refugee's camps of the country with low socioeconomic status. Furthermore, this study has been conducted in rural area with low socioeconomic status as compared to urban area such Kampala and Jinja. In addition, this current study focuses on children age from 2 months to 12 years compared to the previous studies that involve the age range from 6 months to 12 years.

Comparing the prevalence of AKI in malaria against other diseases, the prevalence of AKI reported in this study was comparable with the one reported by Marzuillo et al. [13] among children with diabetes mellitus (43.8%). However, it was significantly higher than in acute gastroenteritis (24.6%), community acquired pneumonia (20.4%), and acute appendicitis (7.4%) [14–16].

### 4.2. Factors Associated with AKI among Children with Severe Malaria

The factors that were significantly associated with AKI among children with severe malaria in this study included caretaker with no formal education or primary level of education, age of child less than 5 years, history of receiving NSAIDs, and moderate to severe anemia. Children aged less than 5 years were 1.8 times more likely to develop AKI compared to those aged 5 years and more (Table 2). Similarly Oshoma et al. [17] in Nigeria found that the median age of children with severe malaria associated to AKI was 3.5 years. Conroy et al. [6] in Uganda found a median age of 3.2 years. This can be explained by the fact that children less than 2 years were shown having a high risk of developing AKI in high transmission area of malaria [6].

In this study, children with caretaker with no formal education or primary level of education were 21 and 4.5 times more likely to develop AKI as compared to those with secondary education (Table 2). This could be because of delayed care seeking behaviors due to less knowledge of child health and development of people with low education levels. However, they were no any identify studies on association about education level of caretakers and AKI.

The present study showed that children with history of receiving NSAIDs were 5.6 times more likely to develop AKI as compared to those with negative history (Table 2). Similarly, a retrospective study conducted in Togolese children by Batte et al. [18] found that 12.1% of children with AKI were on NSAIDs. In Uganda, Conroy et al. [6] found that 5.5% children with severe malaria-associated AKI were on NSAIDs. This can be explained by the fact that NSAIDs are harmful and can induce acute renal failure specifically tubular nephritis in children [19].

The current study showed that children with moderate and severe anemia were 3.1 and 3.8 times more likely to develop AKI (Table 2). Similarly, Batte et al. [18] found that severe anemia was associated with AKI (79.2%). Sabi et al. [17] in India found that 83.3% of children with severe malaria-associated AKI had anemia. Studies have reported that parasite sequestration, hemolysis, and endogenous nephrotoxic (malaria pigment) are frequent etiological factors of severe malaria-associated AKI [20]. Hemolytic anemia can cause AKI by promoting heme pigment related cellular injury and cell death in renal tubules. Several studies have shown that herbal medicine is associated with acute kidney injury [18]. However, in this study, we did not find the association since all participants in this study denied the history of use of herbal medicine.

## 5. Conclusion

There is a high prevalence of acute kidney injury among children with severe malaria in Kiryandongo General Hospital. Acute kidney injury among children with severe malaria was associated with low level of education of caretakers, young age of children (less than 5 years), history of receiving NSAIDs, and anemia (moderate and severe).

## 6. Study Limitations

The cutoff used in this study to define renal impairment (Cr > 3 mg/dL) was chosen in accordance with the WHO recommendations for the definition of AKI in children with severe malaria [21]. However, it is recognized that the WHO criteria in severe malaria underestimate the prevalence of AKI.

## 7. Recommendations

Due to high prevalence of AKI among children with severe malaria, creatinine test should be added among routine investigation of children with severe malaria. The management of severe malaria should include screening for AKI especially in children under five years of age, anemic, and those who have received NSAIDs.

## Figures and Tables

**Table 1:** Baseline sociodemographic characteristics of study participants (*N* = 350).

Sociodemographic characteristics	*N* = 350
Mean age of child in years (SD)	7.0 (3.8)
Age categories of child in years, *n* (%)	
2 months–5 years	109 (31.1)
5 years–12 years	241 (68.9)
Sex of child	
Male	190 (54.3)
Female	160 (45.7)
Mean age of caretaker in years (SD)	28.8 (7.4)
Age categories of caretaker in years, *n* (%)	
15–19	55 (15.7)
20–24	39 (11.2)
25–48	256 (73.1)
Level of education of caretaker, *n* (%)	
No formal education	5 (1.4)
Primary	323 (92.3)
Secondary	22 (6.3)
Mean duration of illness before admission in days (SD)	3.1 (0.93)
Duration of illness before admission in days, *n* (%)	
1-2	63 (18.0)
3 and above	287 (82.0)
Received aminoglycosides, *n* (%)	
No	345 (98.57)
Yes	5 (1.43)
Received NSAIDs, *n* (%)	
No	310 (88.6)
Yes	40 (11.4)
Hyperparasitemia, *n* (%)	
No	83 (23.7)
Yes	267 (76.3)
Mean hemoglobin level (SD)	9.1 (3.36)
Hemoglobin/anemia, *n* (%)	
Normal	125 (35.7)
Mild	44 (12.6)
Moderate	28 (8.0)
Severe/life-threatening	153 (43.7)

**Table 2:** Factors associated with acute kidney injury among children with severe malaria.

Variables	No AKI, *n* (%)	AKI, *n* (%)	cOR (80% CI)	*p*	aOR (95% CI)	*p*
Sex of child						
Male	107 (56.3)	83 (43.7)	1.0		1.0	0.096
Female	76 (47.5)	84 (52.5)	1.4 (0.93–2.17)	0.0998	1.5 (0.93–2.31)	
Age of child in years						0.025
2 months-5 years	44 (40.4)	65 (59.6)	2.0 (1.27–3.19)	0.0026	1.8 (1.07-2.88)	
5-12 years	139 (57.7)	102 (42.3)	1.0		1.0	
Age of caretaker (years)					—	—
15-19	19 (34.6)	36 (65.5)	2.5 (1.33–4.84)	0.0134		
20-29	81 (54.0)	69 (46.0)	1.1 (0.72–1.81)	0.0121		
30-48	83 (57.2)	62 (42.7)	1.0			
Level of education of caretaker						
None	1 (20.0)	4 (80.0)	13.6 (1.22–15.1)	0.0161	21.0 (1.68-261.18)	0.018
Primary	165 (51.1)	158 (49.0)	3.3 (1.17–9.03)	0.0135	4.5 (1.41-14.12)	0.011
Secondary	17 (77.3)	5 (22.7)	1.0		1.0	
Illness duration					—	—
1 to 2 days	35 (55.6)	28 (44.4)	1.0			
3 and above	148 (51.6)	139 (48.4)	1.2 (0.68–2.03)	0.5657		
Received aminoglycosides					—	—
No	181 (52.5)	164 (47.5)	1.0			
Yes	2 (40.0)	3 (60.0)	1.7 (0.27–10.03)	0.5791		
Received NSAIDs						
No	174 (56.1)	136 (43.9)	1.0		1.0	
Yes	9 (22.5)	31 (77.5)	4.4 (2.03–9.57)	<0.0001	5.6 (2.34–13.22)	<0.001
Hyperparasitemia					—	—
No	37 (44.6)	46 (55.4)	1			
Yes	146 (54.7)	121 (45.3)	1.5 (0.91-2.46)	0.209		
Convulsions					—	—
No	114 (53.3)	100 (46.7)	1.0			
Yes	69 (50.7)	67 (49.3)	1.1 (0.72-1.70)	0.6434		
Vomiting					—	—
No	115 (51.1)	110 (48.9)	1.0			
Yes	67 (54.0)	57 (46.0)	0.9 (0.57-1.38)	0.6010		
Diarrhea					—	—
No	147 (52.1)	135 (47.9)	1.0			
Yes	36 (52.9)	32 (47.1)	1.0 (0.57-1.65)	0.9040		
Jaundice					—	—
No	180 (52.5)	163 (47.5)	1.0			
Yes	3 (42.9)	4 (57.1)	1.5 (0.32-6.68)	0.6139		
Hemoglobin/anemia						
Normal	59 (47.2)	66 (52.8)	1.0		1.0	
Mild	34 (77.3)	10 (22.7)	3.4 (1.22-9.45)	0.0037	2.7 (0.92-8.05)	0.070
Moderate	14 (50.0)	14 (50.0)	3.4 (1.59-7.46)	0.0025	3.1 (1.39-6.94)	0.006
Severe/life-threatening	76 (49.7)	77 (50.3)	3.8 (1.73-8.37)	0.0001	3.8 (1.66-8.55)	0.002

## Data Availability

The data used to support this study are available upon request to the corresponding author.

## Abbreviations

AKI: Acute kidney injury
CBC: Complete blood cells
CKD: Chronic kidney disease
DRC: Democratic Republic of Congo
Hb: Hemoglobin
KGH: Kiryandongo General Hospital
NSAIDs: Nonsteroidal anti-inflammatory drugs
SD: Standard deviation
SM: Severe malaria
WHO: World Health Organization.
